# Addressing Implementation Challenges to Digital Care Delivery for Adults With Multiple Chronic Conditions: Stakeholder Feedback in a Randomized Controlled Trial

**DOI:** 10.2196/23498

**Published:** 2021-02-01

**Authors:** Kelly Williams, Sarah Markwardt, Shannon M Kearney, Jordan F Karp, Kevin L Kraemer, Margaret J Park, Paul Freund, Andrew Watson, James Schuster, Ellen Beckjord

**Affiliations:** 1 UPMC Center for High-Value Health Care Insurance Services Division UPMC Pittsburgh, PA United States; 2 Department of Psychiatry College of Medicine-Tucson University of Arizona Tuscon, AZ United States; 3 Community Wellness Consultancy Pittsburgh, PA United States; 4 Consumer Action Response Team of Allegheny County NAMI Keystone Pennsylvania Pittsburgh, PA United States; 5 Department of Surgery UPMC Pittsburgh, PA United States

**Keywords:** telehealth tools, smartphones, remote patient monitoring, care management, mobile phone

## Abstract

**Background:**

Digital tools accessed via smartphones can promote chronic condition management, reduce disparities in health care and hospital readmissions, and improve quality of life. However, whether digital care strategies can be implemented successfully on a large scale with traditionally underserved populations remains uncertain.

**Objective:**

As part of a randomized trial comparing care delivery strategies for Medicaid and Medicare-Medicaid beneficiaries with multiple chronic conditions, our stakeholders identified implementation challenges, and we developed stakeholder-driven adaptions to improve a digitally delivered care management strategy (high-tech care).

**Methods:**

We used 4 mechanisms (study support log, Patient Partners Work Group log, case interview log, and implementation meeting minutes) to capture stakeholder feedback about technology-related challenges and solutions from 9 patient partners, 129 participants, and 32 care managers and used these data to develop and implement solutions. To assess the impact, we analyzed high-tech care exit surveys and intervention engagement outcomes (video visits and condition-specific text message check-ins sent at varying intervals) before and after each solution was implemented.

**Results:**

Challenges centered around 2 themes: difficulty using both smartphones and high-tech care components and difficulty using high-tech care components due to connectivity issues. To respond to the first theme’s challenges, we devised 3 solutions: tech visits (eg, in-person technology support visits), tech packet (eg, participant-facing technology user guide), and tailored condition-specific text message check-ins. During the first 20 months of implementation, 73 participants received at least one tech visit. We observed a 15% increase in video call completion for participants with data before and after the tech visit (n=25) and a 7% increase in check-in completion for participants with data before and after the tech visit (n=59). Of the 379 participants given a tech packet, 179 completed care during this timeframe and were eligible for an exit survey. Of the survey respondents, 76% (73/96) found the tech packet helpful and 64% (62/96) actively used it during care. To support condition-specific text message check-in completion, we allowed for adaption of day and/or time of the text message with 31 participants changing the time they received check-ins and change in standard biometric settings with 13 physicians requesting personalized settings for participants. To respond to the second theme’s challenges, tech visits or phone calls were made to demonstrate how to use a smartphone to connect or disconnect from the internet, to schedule video calls, or for condition-specific text message check-ins in a location with broadband/internet.

**Conclusions:**

Having structured stakeholder feedback mechanisms is key to identify challenges and solutions to digital care engagement. Creating flexible and scalable solutions to technology-related challenges will increase equity in accessing digital care and support more effective engagement of chronically ill populations in the use of these digital care tools.

**Trial Registration:**

ClinicalTrials.gov NCT03451630; https://clinicaltrials.gov/ct2/show/NCT03451630.

## Introduction

Over the last 10 years, ownership and use of smartphones has more than doubled in the United States, from 35% to 81% of the population [1]. One potential benefit of increased access to smartphones is the reduction in health disparities. As smartphone ownership becomes more equitable across socioeconomic categories [1], the use of smartphones provides an opportunity for traditionally underserved or isolated populations to remain connected to health care professionals despite geographic distance or mobility limitations, to quickly receive up-to-date and accurate health education information, and to monitor changes in health conditions using digital health care strategies [2]. In the context of care management teams, remote monitoring platforms provide the opportunity to scale programs, allowing teams of health care professionals and social workers to reach a higher number of individuals living in medically underserved areas [3].

Digital care tools have a growing evidence base, including evidence supporting the effectiveness of such technologies for patients managing chronic conditions. For example, these tools can support individuals with diabetes in lowering hemoglobin A_1c_ levels, improve quality of life and lower number of hospital readmissions for individuals with heart failure [4], improve symptoms and outcomes for individuals with respiratory conditions [5], support better blood pressure control for individuals with hypertension [6], and reduce symptoms of depression [7]. Increased access to and use of digital care strategies has the potential to increase health systems’ ability to achieve the quadruple aim: improving population health, enhancing both patient and provider experience, and reducing costs [8].

Despite increased smartphone use and evidence supporting the benefits of digital care strategies such as remote patient monitoring, biosensors, and wearable devices, barriers to care are ever-present when implementing digital tools on a large scale. Notably, there is a lack of research on the challenges that occur when implementing digital care with traditionally underserved populations and those with high-burden, high-cost medical conditions [9]. A pervasive barrier to the success of digital tools is that individuals may lack confidence in their ability to learn how to use these new tools, which may impact their readiness to engage with such tools [10]. According to recent research on mobile devices and health, over half of Americans are considered to have low digital literacy skills when it comes to using mobile devices [11]. A recent survey of Americans aged above 65 years indicated that although respondents generally had a positive view of technology, they doubted their capacity to learn to use new technology without extra help [12]. In addition, although access to smartphones is becoming more prevalent among all socioeconomic groups, a digital divide still exists in the United States between high- and low-income Americans [13,14], with some research showing that both access and ability may be contributing reasons for why low-income adults may use online health resources less [15,16]. Finally, research has historically focused on remote patient monitoring care effectiveness with older Americans [17] and may not address common challenges faced by younger and other diverse populations.

Overcoming these challenges to realize the full potential of digital care to support the management of chronic conditions and reduce health disparities requires an iterative development approach that includes ongoing consumer and community stakeholder input. We are conducting a large-scale, randomized controlled trial comparing the effectiveness of 3 care management strategies (ie, high-touch, high-tech, and usual care) delivered by a commercial insurance organization for adult Medicaid and Medicare-Medicaid beneficiaries living with multiple chronic conditions. To address the unique needs and challenges experienced by this population and to ensure that our digital interventions are patient-centered and pragmatic, we describe early implementation challenges and our stakeholder-driven process adaptions specific to the digitally delivered chronic disease care management strategy (high-tech care).

## Methods

### Randomized Controlled Trial Overview

The 3 study comparators are approaches to care delivered by a chronic disease care management program and incorporate fundamental, evidence-based components of integrated care models including interdisciplinary care management [18,19], individualized care plans [18,19], chronic disease self-management education or self-care support [8,18,20-24], and linkages to medical/behavioral health and social services [18,25,26]. High-touch is delivered primarily face-to-face, with telephonic support as needed. High-tech is delivered via a remote care management platform. Both high-touch and high-tech participants receive care management for at least 4 months and can continue care for up to 1 year, based on need. Usual care consists of an initial visit and care management for 14 days, which includes connections to condition management support and resources. Care managers receive a weekly worklist denoting individuals who are eligible to be offered participation in the study. To be eligible, individuals must be 21 years or older, have Medicaid or Medicare-Medicaid insurance, have at least 2 chronic conditions, including 1 physical health condition, and have been discharged from a hospital within the past 30 days.

### Introducing Participants to the High-tech Care Strategy

All study participants work, one on one, with a care manager (ie, nurse, social worker, and licensed professional counselor) to create individualized care plans [18,19] centered around chronic disease self-management education, self-care support [8,18,20-24], and to form linkages to medical, behavioral health, and social services [18,25,26]. Participants in the high-tech care management strategy have an initial face-to-face appointment with their care manager and are provided with a preconfigured iPhone that allows for care to continue digitally via a remote monitoring platform. We provide iPhones to participants to ensure access to smartphones, and the cost of cellular data is not a barrier to participation. At the initial appointment, the care manager explains 2 key components of the remote monitoring platform that the participant will use on a regular basis: video visits (eg, video conferencing between patients and care managers) and condition-specific text message check-ins. The remote monitoring platform facilitates video conferencing (eg, video visits) between participants and their care manager. Moreover, as our study population has multiple physical and behavioral health conditions, condition-specific check-in questions are sent via text messages to each participant at varying intervals (eg, daily, weekly, or biweekly) based on their condition(s). Check-ins allow care managers to monitor diverse participant needs, symptoms, or condition exacerbations, including specific biometric readings such as pulse, blood glucose level, weight, and blood pressure.

### Stakeholder Engagement and Feedback Processes

At the onset of implementation, the study team was acutely aware of the need for continued stakeholder engagement and feedback to promote effective high-tech care implementation. On the basis of the Patient-Centered Outcome Engagement principles [27], we developed 4 mechanisms (Textbox 1) to capture feedback and input from various stakeholder groups. Patients, care managers, and clinical leadership all provided key insights and observations related to technology challenges that care managers or participants experienced during high-tech care implementation. This stakeholder input was collated from the study support log, the case interview log, and the implementation meeting minute log (Textbox 1). Feedback was iteratively reviewed by the study and the clinical team. Stakeholder input was organized by topic and content to understand early stage implementation challenges. Topics were then reviewed by the study team and organized into 2 major thematic categories; themes were reviewed with key stakeholders for validation.

Using information from these 3 feedback mechanisms, we developed solutions to the identified technology-related challenges. Solutions were discussed, refined, and implemented with input from the study team, care managers, and clinical leadership. Solutions were also vetted through the Patient Partners Work Group. A work group of patient partners, who have similar characteristics and lived experiences similar to those experienced by our study population, was established through a collaboration with the National Alliance on Mental Illness Southwestern Pennsylvania’s Consumer Action Response Team. Patient partner feedback was tracked in the Patient Partners Work Group log (Textbox 1).

**Mechanisms to capture stakeholder feedback on challenges and/or solutions.**

Data source and information collected and provided:

- Study support log
  Study team created a study-specific, toll-free, hotline staffed during office hours
  Hotline supports care managers and participants with study-related questions or challenges
- Implementation meeting minutes
  Study team meets with clinical leadership weekly and meets monthly with all care managers
  Meetings provide a time and space for care managers and their clinical leadership to voice implementation challenges and to strategize potential solutions
- Case interview log
  Semistructured interviews were conducted with care managers to identify technology-related challenges, participants experience, and workflow impacts
- Patient Partners Work Group log
  Study team meets regularly with the work group to discuss high-tech care implementation
  The work group provides feedback on materials and solutions supporting high-tech care engagement/implementation

### Understanding Process Modifications: Sources of Information

In order to assess a change in participants’ abilities to overcome technology-related challenges, we analyzed intervention engagement outcomes that may have been impacted by stakeholder-driven implementation solutions. We reviewed the following 3 sources of engagement data pertaining to care activity from April 23, 2018, to December 31, 2019: (1) the participants’ ability to complete a video visit as defined by answering the video call from their care manager, (2) the participants’ ability to answer condition-specific text message check-ins, as defined by receiving a check-in via text message and submitting all answers to condition-specific questions, and (3) participant responses to exit survey questions sent via the remote monitoring platform. All pre- and postdata presented are based on the first in-person technology support visit completed by the participant.

## Results

### Overview

Stakeholders reported challenges centered around 2 major themes: (1) difficulties using basic functionalities of the smartphone and high-tech care components and (2) difficulties using high-tech care components due to cellular reception and internet connectivity issues. Approximately 500 study hotline phone calls, about technology-specific challenges, were made by 129 participants and 32 care managers to the study team, and the calls were tracked in the study support log. Feedback was also provided during clinical leadership meetings (n=82), monthly care management staff meetings (n=16, tracked via implementation meeting minutes), and semistructured interviews with care managers (n=4, tracked via case interview log). For each thematic challenge, we present: (1) specific stakeholder feedback that leads to solution development, (2) the description of the stakeholder-driven solutions as they are a direct result of stakeholder feedback, and (3) changes in participant engagement data after solution implementation.

### Theme 1 Challenge: Smartphone and High-tech Care Digital Component Use

With support from care managers and clinical leadership, the study team focused on common functionality challenges experienced by high-tech care participants and devised 3 main solutions: (1) tech visits (technology support visits), (2) a tech packet (participant-facing technology user guide), and (3) tailored condition-specific text message check-in. Table 1 displays stakeholder feedback regarding the participants’ experiences when using the smartphone and high-tech care components.

**Table 1:** Sources of information and solutions: utilizing functionalities of the smartphone and high-tech components.

Data source	Information provided	Informed solution
Case interview log and study support log	Care managers concerned about time spent teaching participants basic smartphone functionalities Specific technology challenges faced by participants include screen pressure difficulties, home screen navigation, phone charging, text message access, including opening condition-specific check-ins, and phone volume manipulation	Tech visits and tech packet Tech packet
Implementation meeting minutes	Clinical leadership interpreted technology-education time concerns as a workflow issue in which care managers had to make up time to ensure participant clinical care needs were met	Tech visits and tech packet
Study support log	Smartphone factory resets and reconfiguration were time consuming for care managers Smartphone volume manipulation and battery power were 2 participant challenges that resulted in missed high-tech care video visits and condition-specific check-ins Check-in assignments were sometimes automatically scheduled at inconvenient times for participants (ie, work, school or sleeping hours) Biometric check-in settings were automatically standardized for each participant	Tech visits and tech packet Tech packet Tailored condition-specific check-ins Tailored condition-specific check-ins

#### Technology Support Visit (Tech Visit) Solution for Theme 1: Description and Changes in Engagement

Tech visits are structured to allow a study team member to assist participants with time-consuming digital literacy challenges, either at the participant’s home or at a community location. Tech visits do not replace initial face-to-face training that care managers provide to high-tech care participants; rather, it is a form of supplemental training to ensure that participants are able to use their smartphone to receive care and to reduce the time care managers spend on high-tech care training during the initial appointment. Participants are selected for tech visits if (1) they have called the study hotline multiple times with issues that could not be completely resolved, (2) a care manager is unable to connect with the member due to technology challenges, and (3) clinical supervisors believe a participant’s level of digital literacy requires a substantial amount of care manager’s time.

**Figure 1:**
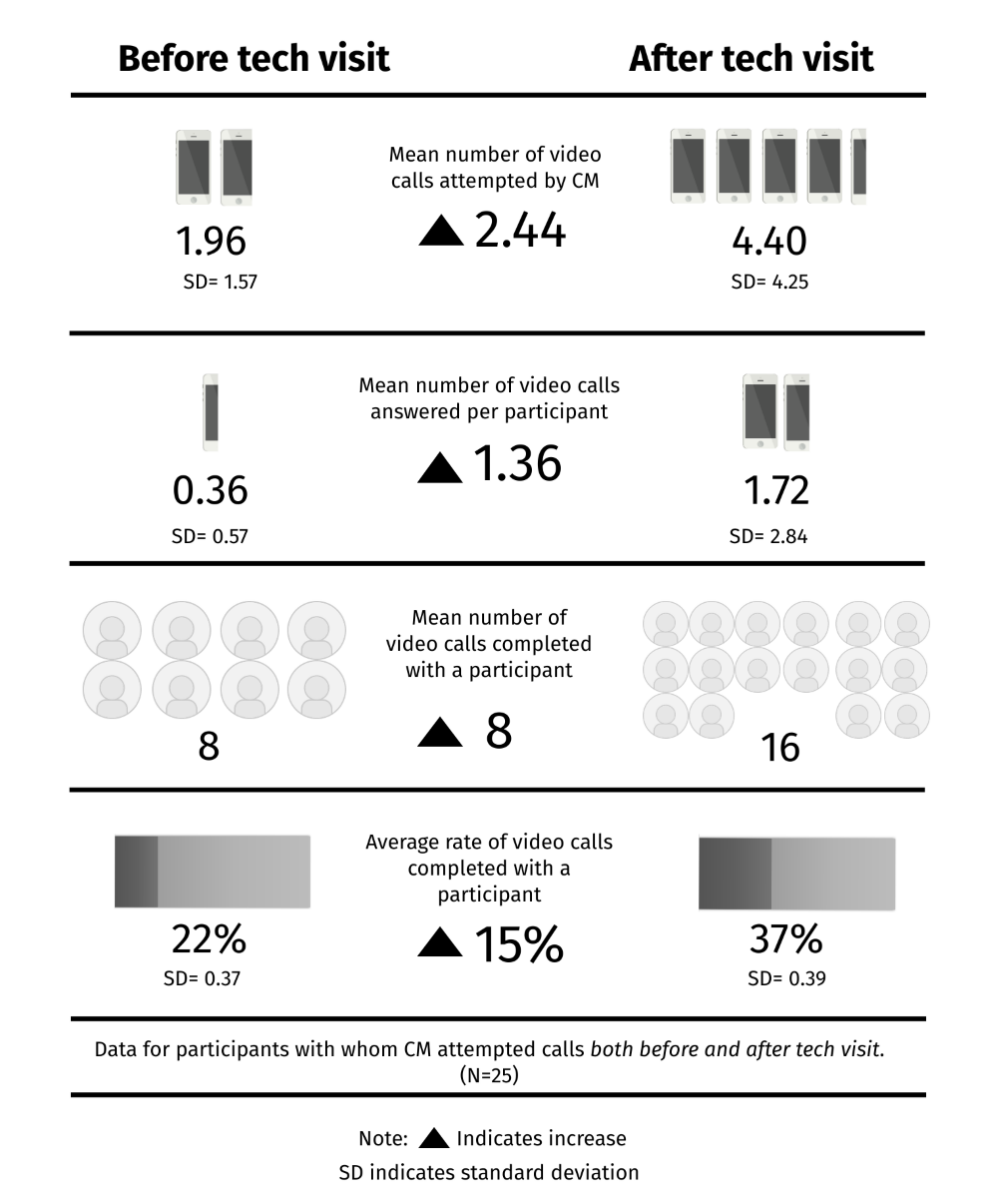
Video call data for participants with completed pre- and postdata. CM: care manager.

During the first 20 months of implementation, 73 participants received at least one tech visit. Before the tech visit, 23% (17/73) of the participants completed a video call with their care manager. Within 30 days of a tech visit, the average rate for video call completion increased to 51% (n=33). In total, 21 members, who had never been able to connect with their care manager via video calls, before the tech visit completed a video call after receiving the tech support. Of the 73 participants, 25 (34%) had video call data for both before and after the tech visit. For these participants, the average rate of completed calls increased by 15% after the completion of a tech visit (Figure 1).

**Figure 2:**
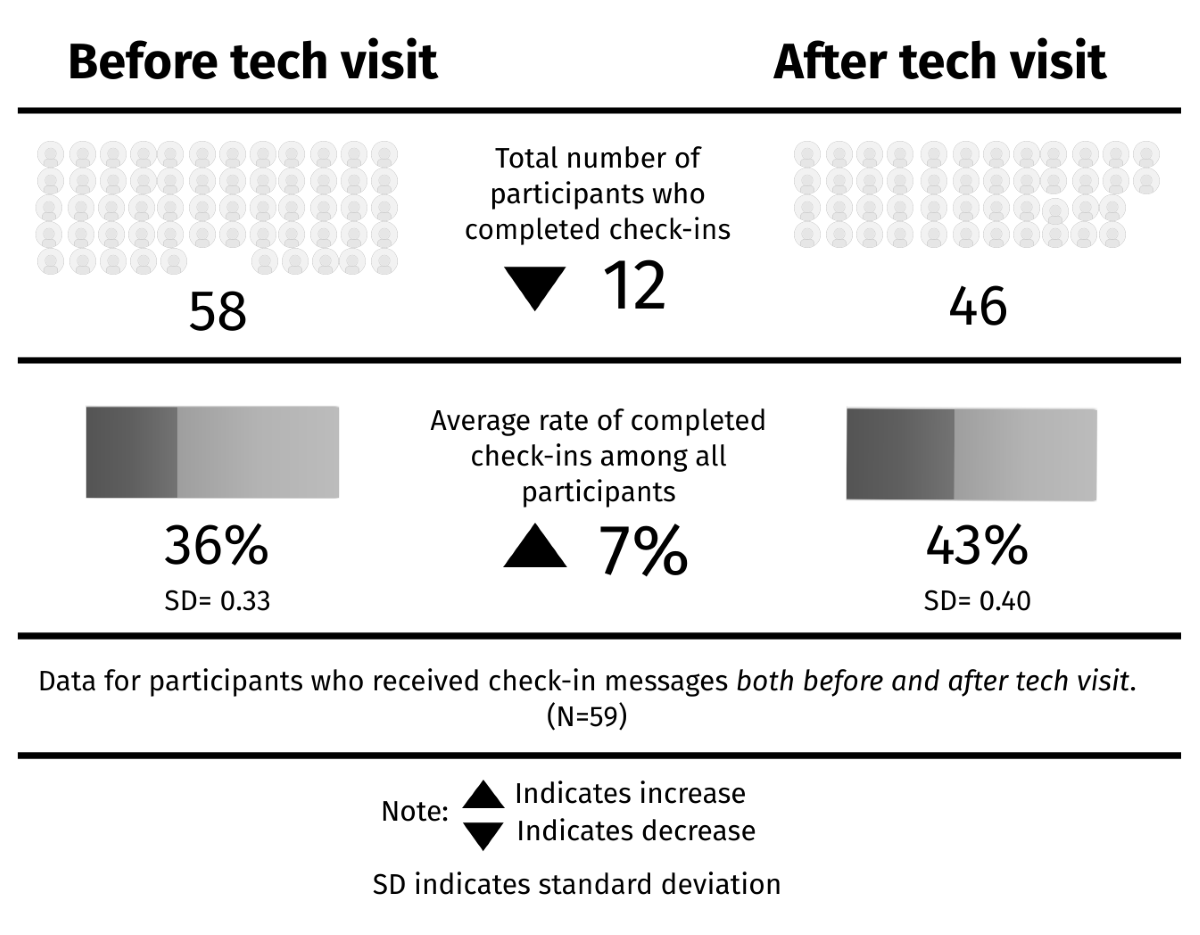
Check-in data for participants with completed pre- and postdata.

Specific to condition-specific check-ins sent via text message, most participants completed at least one check-in on their own before their first tech visit (60/73, 82%), with an average rate of 36% (n=59). Within 30 days of a tech visit, the average rate for engagement with check-ins increased to 43% (n=59). Two participants completed check-ins after receiving support, who had never completed a check-in prior. Conversely, 15 participants completed check-ins before receiving support but never completed a check-in after the visit. Of the 73 participants, 59 (81%) had check-in data both before and after the tech visit. For these participants, the average rate of completed check-ins increased by 7% after the completion of a tech visit (Figure 2).

#### Participant-Facing Technology User Guide (Tech Packet) Solution for Theme 1: Description and Changes in Engagement

**Figure 3:**
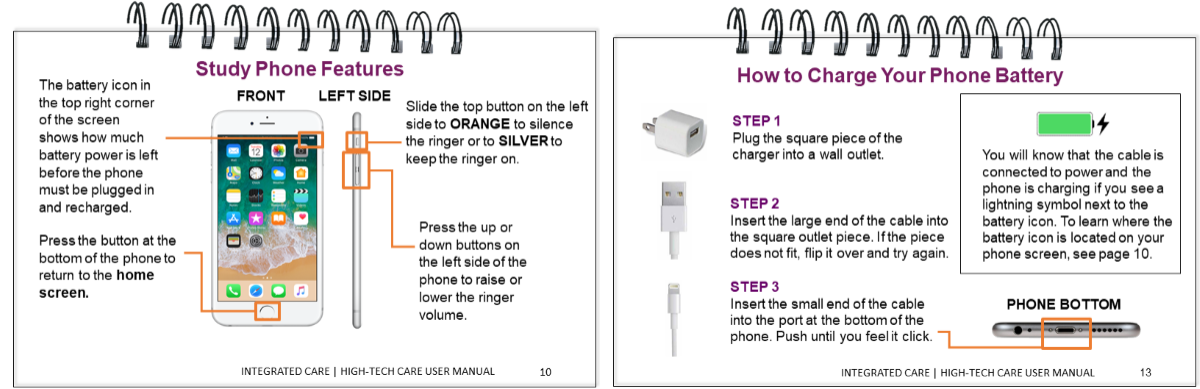
Tech packet additions based on study support log.

**Figure 4:**
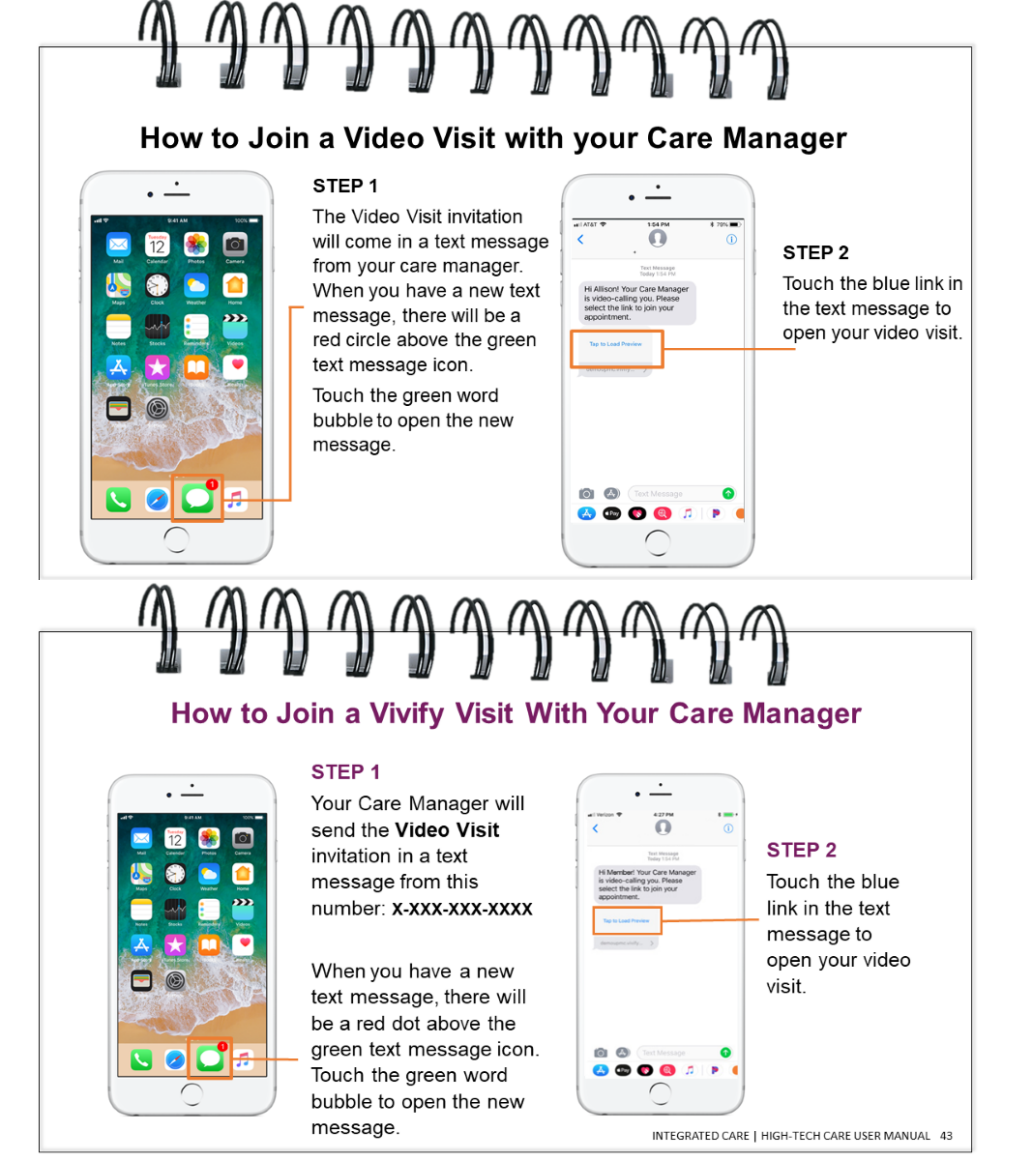
Tech packet updates pre- and postpatient Partners Work Group feedback.

The participant-facing technology user guide (tech packet) is an educational resource for participants learning how to use the smartphone and high-tech care digital components. The tech packet outlines key smartphone functions (ie, how to answer a phone call, how to navigate to the *home screen*, how to open a text message, etc) and high-tech digital care components (ie, how to answer a video call and how to respond to a condition-specific check-in). Care managers give the tech packet to participants during the initial appointment when the participant is randomized into high-tech care; care managers explain the tech packet and have participants practice key functions that they will use throughout their care. Figure 3 shows 2 pages from the tech packet that were developed in response to specific challenges reported in the study support log (Table 1). Feedback from our patient partners, members of the Patient Partners Work Group, was collected over 2-hour-long meetings. Table 2 displays the Patient Partners Work Group feedback on the solution and details on how their feedback was incorporated, and Figure 4 presents a visual example of feedback incorporation. The tech packet is updated regularly based on participant, patient partner, care manager, and clinical leadership feedback.

**Table 2:** Patient Partners Work Group technology user guide feedback.

Work group feedback	Examples of feedback	Feedback incorporated
Use less abbreviations/ jargon as these are difficult to follow	App has many meanings to different participants Remote monitoring may have a negative connotation for participants	Addition of definitions page and spelling out abbreviations: app to application Replacement of technical jargon: Check-ins with your care manager instead of remote monitoring
Provide an easy *start point* for each section; assume the lowest level of digital literacy when creating the instructions	Each section begins on the smartphone home screen, which assumes participants can find the home screen	Addition of instructions on how to navigate to home screen at the beginning and end of each section
Dexterity and pressure difficulties may be a concern	Press, click, touch, and open were used interchangeably	Used touch for screen actions and press for the home screen button to distinguish amount of pressure to be applied
Highlight important contact information associated with care management and study activities	Participants may not be able to distinguish who is sending a text message, and some may be concerned about the legitimacy of messages	Stated explicitly messages from the remote monitoring platform come from the same phone number each time and make the number visible on all sections Displayed the study hotline number frequently
Participants can be difficult to reach	Participants may not be reached during business hours	Included a section on how to set up and check a voicemail box

**Figure 5:**
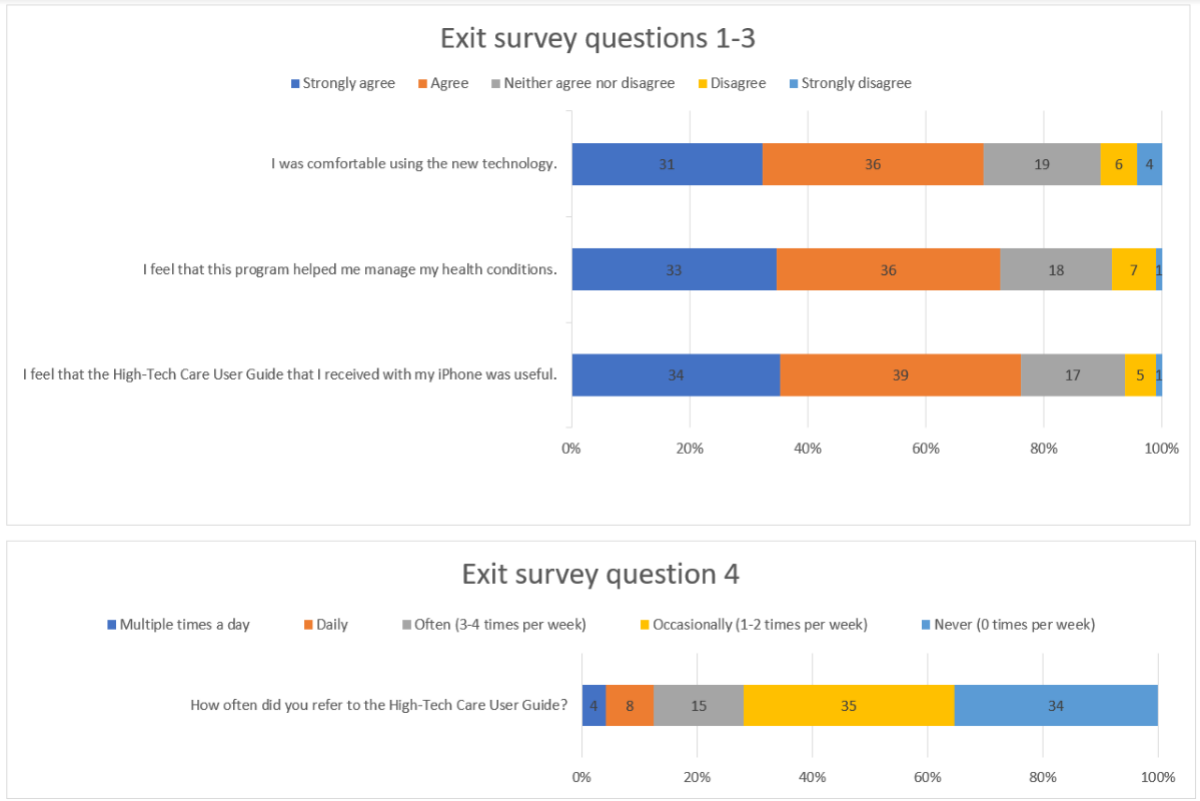
High-tech care exit survey (96/179, 53.6%).

We distributed the tech packets to 379 participants. Of the individuals who received a packet, 179 participants were eligible (eg, completed high-tech care before January 1, 2020) to receive the high-tech care exit survey. Of the 179 participants, 96 responded to the survey; 73 respondents strongly agreed or agreed that the tech packet was useful and 62 actively used the guide at least 1 to 2 times a week during care. See Figure 5 for detailed exit survey results.

#### Check-in Tailoring Solution for Theme 1: Description and Impact

Not all challenges presented to the study team were solved through tech visits and the tech packet. To support participants in completing their condition-specific check-ins, the study team adapted their workflow to allow for a modification of when check-ins are assigned (ie, day of the week and time) based on participant preference. In total, 31 participants requested to change the day/time when check-in text messages were to be received. Second, as all biometric check-in settings (eg, normal boundary parameters for pulse, blood glucose level, weight, and blood pressure) were standardized across participants by default, the study team allowed individual check-in settings to be modified based on the agreement of a participant’s primary medical provider. For 13 participants, the primary medical provider requested biometric setting changes to reflect the participant’s *normal* range; this modification allows care managers to better track if biometric readings fall outside the participant’s expected range. Textbox 2 describes specific cases in which check-in assignments or biometric settings were modified.

**Condition-specific check-in tailoring examples.**

- Check-in assignments modified based on participant preferences
  Participant rescheduled their diabetes check-in for the morning, based on when their medical provider had instructed them to check their glucose
  Participant rescheduled their check-in assignment to the day they are off work
- Biometric settings modified based on provider preferences
  Primary medical provider verified that the participant takes glucose readings before taking insulin and requested setting alerts to be set at 270 or above
  Primary medical provider approved to change a participant’s blood pressure settings; allowing notification to only send to the care manager when the participant is out of their expected range >170/100 or <90/60

### Theme 2 Challenge: Limited Cellular Reception and Internet Connectivity

It was reported, by both care managers and participants themselves, that participants were having difficulties using high-tech care digital components (eg, video visits and condition-specific check-ins) due to limited cellular reception and internet connectivity. Depending on the circumstances, solutions include (1) participant education on how to connect a smartphone to the internet, (2) schedule video visits or condition-specific check-ins when the participant is in a location with cellular service or internet, and (3) participant education on how to disconnect from the internet. Table 3 displays the sources of information that led the study team to become aware of the challenge participants experienced with connectivity.

**Table 3:** Sources of information and solutions: understanding limited cellular reception and intervention connectivity.

Data source	Information provided	Informed solution
Study support log and implementation meeting minutes	Server error messages reported by participants preventing check-in messages from opening	Education on connecting a smartphone to the internet
Study support log and implementation meeting minutes	No internet at the home preventing digital tool use No cellular reception at home prevented digital tool use	Scheduling video visits/check-ins when participant has access to cellular service or internet
Study support log and implementation meeting minutes	Poor internet service at home chosen as default connection method prevented digital tool use Poor internet service in community chosen as default connection method prevented digital tool use	Education on disconnecting from the internet

#### Solutions for Theme 2: Description and Changes in Engagement

Care managers communicate with each participant regarding the best way to provide care, given limited cellular reception and internet connectivity. All 3 connectivity solutions are addressed via a tech visit or phone call. Providing education on connecting a smartphone to the internet is often a solution for participants receiving a *server error* message when attempting to open condition-specific check-ins. Participants were advised to connect to the home internet in case of a bandwidth issue. Scheduling video visits and condition-specific check-ins at specific times affords participants the knowledge that they will have access to cellular service or internet and has been another viable solution. For example, one participant rescheduled their condition-specific check-ins to days of the week when a routine visit was set with family members who have internet access or to business hours as their employer offers internet access. Finally, providing education to participants on how to disconnect from the internet can support video visits or check-in completion. For example, participants are instructed to disconnect weak internet connections, such as home connections or public connections, when they have strong cellular service.

## Discussion

### Principal Findings

This paper highlights the impact of stakeholder-driven solutions on early implementation challenges specific to a digital care strategy. To support participants in engaging with their smartphone and high-tech care digital components (theme 1 challenge), 3 main solutions were implemented (eg, in-person technology support visits, participant-facing technology user guides, and tailored condition-specific check-ins). For participants who received an in-person technology support visit, we saw an overall increase in engagement with video calls and condition-specific check-ins. For participants who received the participant-facing technology user guide and completed both high-tech care and the exit survey, we found that most used the tech packet while receiving care and or believed it was useful. Finally, condition-specific check-ins were tailored for participants to support engagement and meet their primary medical provider’s care goals. To support participants experiencing difficulties engaging in high-tech care due to limited cellular reception or internet connectivity (theme 2 challenges), 3 solutions were devised and implemented as needed to support engagement in the program (eg, education on both connecting and disconnecting a smartphone to the internet, scheduling high-tech care video visits or condition-specific check-ins at times when the participant is in a physical location that allows connectivity to occur).

Our findings suggest that concurrent stakeholder feedback has the potential to increase implementation success; therefore, it is pivotal to provide stakeholders with multiple and continuous avenues for communicating challenges to the study team. Furthermore, the results stress the importance of working collaboratively with stakeholders early in the implementation of digital interventions to design scalable solutions such as educational materials (tech packet) and activities (tech visits and telephonic support) and refine condition-specific check-ins that suit the specific needs of the patient and their primary medical provider. Moreover, although measuring the success of solutions created during implementation is not always preplanned, early results indicate positive changes in participant technology engagement after tech visits are implemented. Our positive trends in engagement highlight the need for earlier identification of patients who require tech visits to promote early engagement, reduce demoralization, and potentially achieve earlier clinical benefits. Understanding the nuanced challenges of delivering interventions and engaging patients—as well as how to create effective solutions—will advance the efficiency and reach of digital care.

### Comparison With Prior Work

Current digital care literature focuses on either how tool engagement impacts desired health outcome(s) [6] or defining tool use metrics [28,29]. Processes and solutions to overcome tool utilization barriers are underdeveloped topics in the field that has implications for replication and scaling. One systematic review of digital mental health interventions targeting college students found that of the 89 studies, 45 reported outcomes focused on usability and acceptability (many with low rates of response) and only 2 studies reported on feasibility [30]. It is critical to expand knowledge centered on how to design and adapt implementation processes in order for digital care teams to be equipped with the right knowledge and resources to best overcome challenges to digital care provision for chronically ill and low-income populations. Providers delivering care digitally must understand and be able to adequately address patient-specific barriers to using digital care tools before patients can engage in the evidence-based tool functions and work toward improved health outcomes.

Our work is an important addition to the discussion on digital care provision as we provide a systematic framework for how digital care providers can work with stakeholder groups to identify and address care delivery implementation challenges. Although most research on remote patient monitoring focuses on single-diagnosis care for older Americans, our intervention targets adults aged 21 years and older who are managing multiple chronic physical and or behavioral health conditions [6]. Our findings expand knowledge beyond the traditional populations included in digital health research.

Finally, as participants in this research are exclusively eligible for Medicaid, our work promises to reduce health disparities by improving access to digitally delivered evidence-based care management for low-income patients. Supporting consumer adoption of digital health tools is one way to both support patients in their management of chronic conditions and the ethical imperative of reducing health disparities. However, realizing the full potential of digital tools to positively impact health disparities requires continued work on understanding the ways to best support traditionally underserved populations to use digital tools (and how to design those tools and their implementation protocols to meet diverse consumer needs).

### Limitations

Although this study contributes novel stakeholder-driven solutions to stakeholder-reported implementation challenges that affect participants’ engagement in a digital care intervention, there are several limitations. Data collected via the high-tech care exit survey may be limited due to a nonresponse bias, as participants who completed the survey had to be able to access the check-in and be willing to complete the survey after completing all care goals. However, our current response rate of 54% indicates that this bias may be less salient [31]. In addition, we were not able to control for additional factors such as in-home caregiver support, improvement in health conditions, or time participating in care that may have also impacted a participant’s ability to overcome the specific challenges that our solutions were designed to target. However, as our solutions were codesigned with key stakeholders and our ability to review pre- and postsolution data, it is reasonable to assume that our solutions influenced positive trends in high-tech care engagement. We also acknowledge that our provision of a smartphone to all study participants may be perceived as a potential barrier to scalability. However, it is important to understand engagement-related challenges for individuals with varying levels of experience with such technology and provide the same phone to all study participants allows us to understand how heterogeneity in technology comfort/experience manifests over the course of the intervention. For future efforts, care managers can and do support individuals with the procurement of a government-issued smartphone that has similar functionality to the phone provided for this study; thus, our findings related to technology engagement can likely be generalized beyond the scope of this study.

### Conclusions

To better understand digital care barriers specific to a patient population or care program, it is critical to develop and employ methods for obtaining feedback from key stakeholders before and during implementation. Key stakeholders may include care providers and implementation teams, digital tool creators, and community organizations that represent the population of interest and patients. Our stakeholder-informed solutions include in-person tech visits, a tech packet detailing how to access and use key technology components, along with the tailoring of digital care components to meet both patient and provider needs. Using high-tech care exit survey responses and remote monitoring engagement data, we measured the impact of our solutions and continued to improve high-tech care delivery. As solutions to challenges develop, detailed tracking of their implementation may positively impact patient engagement with digital tools and ultimately show increased participation in care resulting in improved health outcomes and reductions in health outcome disparities.

## Abbreviations

PCORI: Patient-Centered Outcomes Research Institute
