# Correction: Addressing Implementation Challenges to Digital Care Delivery for Adults With Multiple Chronic Conditions: Stakeholder Feedback in a Randomized Controlled Trial

**DOI:** 10.2196/27996

**Published:** 2021-02-26

**Authors:** Kelly Williams, Sarah Markwardt, Shannon M Kearney, Jordan F Karp, Kevin L Kraemer, Margaret J Park, Paul Freund, Andrew Watson, James Schuster, Ellen Beckjord

**Affiliations:** 1 UPMC Center for High-Value Health Care Insurance Services Division UPMC Pittsburgh, PA United States; 2 Department of Psychiatry College of Medicine-Tucson University of Arizona Tucson, AZ United States; 3 Center for Research on Health Care Division of General Internal Medicine University of Pittsburgh School of Medicine Pittsburgh, PA United States; 4 Community Wellness Consultancy Pittsburgh, PA United States; 5 Consumer Action Response Team of Allegheny County NAMI Keystone Pennsylvania Pittsburgh, PA United States; 6 Department of Surgery UPMC Pittsburgh, PA United States

In “Addressing Implementation Challenges to Digital Care Delivery for Adults With Multiple Chronic Conditions: Stakeholder Feedback in a Randomized Controlled Trial” (JMIR Mhealth Uhealth 2021;9(2):e23498) the authors noted one error.

In the originally published article, the affiliation for author Kevin L Kraemer:

Center for Research on Health Care, Division of General Internal Medicine, University of Pittsburgh School of Medicine, Pittsburgh, PA, United States

was inadvertently removed and replaced by the affiliation:

Department of Psychiatry, College of Medicine-Tucson, University of Arizona, Tuscon, AZ, United States

In the originally published article, the full list of authors and affiliations was listed as follows:

Kelly Williams^1^, MPH, PhD; Sarah Markwardt^1^, MID; Shannon M Kearney^1^, CPH, MPH, DrPH; Jordan F Karp^2^, MD; Kevin L Kraemer^2^, MD; Margaret J Park^3^, MDiv; Paul Freund^4^, MEd; Andrew Watson^5^, FACS, M. Litt, MD; James Schuster^1^, MBA, MD; Ellen Beckjord^1^, PhD, MPH

^1^UPMC Center for High-Value Health Care, Insurance Services Division, UPMC, Pittsburgh, PA, United States

^2^Department of Psychiatry, College of Medicine-Tucson, University of Arizona, Tuscon, AZ, United States

^3^Community Wellness Consultancy, Pittsburgh, PA, United States

^4^Consumer Action Response Team of Allegheny County, NAMI Keystone Pennsylvania, Pittsburgh, PA, United States

^5^Department of Surgery, UPMC, Pittsburgh, PA, United States

This has been corrected to:

Kelly Williams^1^, PhD, MPH; Sarah Markwardt^1^, MID; Shannon M Kearney^1^, DrPH, MPH, CPH; Jordan F Karp^2^, MD; Kevin L Kraemer^3^, MD; Margaret J Park^4^, MDiv; Paul Freund^5^, MEd; Andrew Watson^6^, FACS, MLitt, MD; James Schuster^1^, MBA, MD; Ellen Beckjord^1^, PhD, MPH

^1^UPMC Center for High-Value Health Care, Insurance Services Division, UPMC, Pittsburgh, PA, United States

^2^Department of Psychiatry, College of Medicine-Tucson, University of Arizona, Tucson, AZ, United States

^3^Center for Research on Health Care, Division of General Internal Medicine, University of Pittsburgh School of Medicine, Pittsburgh, PA, United States

^4^Community Wellness Consultancy, Pittsburgh, PA, United States

^5^Consumer Action Response Team of Allegheny County, NAMI Keystone Pennsylvania, Pittsburgh, PA, United States

^6^Department of Surgery, UPMC, Pittsburgh, PA, United States

The correction will appear in the online version of the paper on the JMIR Publications website on February 26, 2021, together with the publication of this correction notice. Because this was made after submission to PubMed, PubMed Central, and other full-text repositories, the corrected article has also been resubmitted to those repositories.

